# Inhibition of Neuronal p38α, but not p38β MAPK, Provides Neuroprotection Against Three Different Neurotoxic Insults

**DOI:** 10.1007/s12031-014-0372-x

**Published:** 2014-07-11

**Authors:** Bin Xing, Adam D. Bachstetter, Linda J. Van Eldik

**Affiliations:** 1 Sanders-Brown Center on Aging, University of Kentucky, Lexington, KY 40536 USA; 2 Department of Anatomy and Neurobiology, University of Kentucky, Lexington, KY 40536 USA; 3 101 Sanders-Brown Building, 800S. Limestone Street, Lexington, KY 40536-0230 USA

**Keywords:** p38 MAPK, Glutamate, Sodium nitroprusside, Oxygen-glucose deprivation, Neuroprotection, Kinase inhibitor

## Abstract

The p38 mitogen-activated protein kinase (MAPK) pathway plays a key role in pathological glial activation and neuroinflammatory responses. Our previous studies demonstrated that microglial p38α and not the p38β isoform is an important contributor to stressor-induced proinflammatory cytokine upregulation and glia-dependent neurotoxicity. However, the contribution of neuronal p38α and p38β isoforms in responses to neurotoxic agents is less well understood. In the current study, we used cortical neurons from wild-type or p38β knockout mice, and wild-type neurons treated with two highly selective inhibitors of p38α MAPK. Neurons were treated with one of three neurotoxic insults (L-glutamate, sodium nitroprusside, and oxygen-glucose deprivation), and neurotoxicity was assessed. All three stimuli led to neuronal death and neurite degeneration, and the degree of neurotoxicity induced in wild-type and p38β knockout neurons was not significantly different. In contrast, selective inhibition of neuronal p38α was neuroprotective. Our results show that neuronal p38β is not required for neurotoxicity induced by multiple toxic insults, but that p38α in the neuron contributes quantitatively to the neuronal dysfunction responses. These data are consistent with our previous findings of the critical importance of microglia p38α compared to p38β, and continue to support selective targeting of the p38α isoform as a potential therapeutic strategy.

## Introduction

Mitogen-activated protein kinase (MAPK) pathways are pivotal in linking stimuli to cellular responses. The involvement of MAPK pathways in many stress- and disease-induced responses throughout the body has heightened the interest to develop selective small molecule kinase inhibitors to modulate these signal transduction pathways. For example, the p38 branch of the MAPK family is a well-established therapeutic target for diseases with inflammation as a common mechanism. In the central nervous system (CNS), most studies of p38 function have focused on p38 in glia and its role in aberrant proinflammatory responses in acute and chronic neurodegenerative conditions (for reviews, see Bachstetter & Van Eldik 2010; Correa & Eales 2012). Much less is known about the relationship between neuronal p38 and CNS pathophysiology. In addition, whether the two major p38 isoforms in the CNS, p38α and p38β, play similar or distinct roles in neuronal responses to pathological stimuli is a major unanswered question.

Investigations to define the relative importance of neuronal p38α and p38β in stress-induced neuronal responses have been hampered by a lack of specific reagents. Mice with a genetic knockout of the p38β gene (p38β knockout (KO)) are healthy and fertile (Beardmore et al. 2005; O'Keefe et al. 2007), and therefore are a useful reagent to test the involvement of the p38β isoform in particular cellular functions. However, a similar approach cannot be taken with p38α knockout mice because these mice are embryonic lethal (Adams et al. 2000; Allen et al. 2000; Mudgett et al. 2000; Tamura et al. 2000). In addition, many small molecule p38 inhibitors such as the commercially available SB203580 compound do not distinguish between p38α and p38β, and actually react with a number of other cellular targets, including thromboxane synthase (Borsch-Haubold et al. 1998), cyclooxygenases (Borsch-Haubold et al. 1998), c-Raf (Hall-Jackson et al. 1999), and other kinases (Clerk & Sugden 1998; Lali et al. 2000; Godl et al. 2003; Bain et al. 2007). While one might assume that the effects of SB203580 are dependent on p38α, this assumption has not been rigorously tested with p38α- and p38β-specific reagents.

We recently reported (Watterson et al. 2013) the development of two highly specific small molecule p38α inhibitors, termed MW-108 and MW-181. The high level of selectivity of the inhibitors was demonstrated by large-scale kinome activity screens, functional GPCR agonist and antagonist assays, and cellular target engagement analyses. MW-108 targets a single kinase, p38α, and does not cross-react with p38β. MW-181 inhibits p38α, and has weaker cross-reactivity with p38β. The availability of these p38α inhibitors, along with the p38β KO mouse, provided us the opportunity to directly test the contribution of neuronal p38α and p38β in neurodegenerative responses to specific toxic stimuli.

The goal of the current study was to determine whether neuronal p38α or p38β is important for neurotoxic responses induced by three clinically relevant insults: L-glutamate (excitotoxicity), sodium nitroprusside (SNP; a nitric oxide donor), and oxygen-glucose deprivation (OGD; hypoxia ischemia). We chose these three neurotoxic insults because there is precedent for p38 playing a role in neurotoxicity responses induced by these agents (Kawasaki et al. 1997; Lin et al. 2001; Legos et al. 2002; Chen et al. 2003; Cao et al. 2004; Pi et al. 2004; Tabakman et al. 2004; Guo & Bhat 2007; Molz et al. 2008; Strassburger et al. 2008; Li et al. 2009; Lu et al. 2011). We used primary cortical neurons from wild-type (WT) and p38β global KO mice to determine if deletion of p38β affected the neuronal damage responses. To test the contribution of p38α to the neurotoxic responses and to determine if targeting a single kinase was neuroprotective, we treated WT mouse neurons with the neurotoxic agents in the presence of our p38α inhibitors MW-181 and MW-108 (Watterson et al. 2013). Consistent with our previous findings of a distinct role for p38α and p38β in microglia upon inflammatory insult (Xing et al. 2011; Xing et al. 2013), we report here that the absence of p38β in cortical neurons does not suppress the neurotoxic responses to any of the three insults. However, selective inhibition of p38α in neurons not only reduces cell death but also reduces the neurite damage in the surviving neurons. These results demonstrate the importance of the neuronal p38α isoform in neurotoxicity induced by multiple disease-relevant insults.

## Materials and Methods

### Ethics Statement

All mouse experiments were conducted in accordance with the principles of animal care and experimentation in the Guide for the Care and Use of Laboratory Animals. The Institutional Animal Care and Use Committee of the University of Kentucky approved the use of animals in this study (protocol #2010-0615).

### Reagents

L-glutamate (Cat. no. G1251) and SNP (Cat. no. 228710) were obtained from Sigma-Aldrich. The highly selective p38α inhibitors MW01-10-181SRM (MW-181) and MW01-11-108SRM (MW-108) were synthesized and characterized as described (Watterson et al. 2013). Stock solutions of the inhibitors were prepared in sterile 0.9 % NaCl.

### Animals

The p38β global KO mice were generated as described (O'Keefe et al. 2007), and a colony bred and maintained at University of Kentucky. C57BL/6 mice were purchased from Harlan Laboratories. The p38β gene KO was confirmed by Transnetyx, Inc (Cordova, TN, USA).

### Determination of p38 Isoform RNA Levels

The levels of expression of p38α, β, δ, and γ RNA were determined as previously described (Xing et al. 2013). Briefly, RNA was isolated from primary cortical neuron cultures using RNeasy minicolumns with on-column DNase treatment (Qiagen), and RNA quantity and quality were determined by measuring the A_260_/A_280_ ratio by NanoDrop (Thermo Scientific). Reverse transcription (RT) was done with a High Capacity cDNA Reverse Transcription Kit (Applied Biosystems, Cat. no. 4368814), with no template and no RT controls included. Real-time PCR was done with the TaqMan Gene Expression assay kit (Applied Biosystems) on a ViiA 7 Real-Time PCR System (Applied Biosystems). The following TaqMan probes (Applied Biosystems) were used: p38α (MAPK14, Mm00442507_m1), p38β (MAPK11, Mm00440955_m1), p38δ (MAPK13, Mm00442488_m1), p38γ (MAPK12, Mm00443518_m1), and 18S rRNA (Hs99999901_s1). Relative gene expression was calculated by the 2^−ΔΔCT^ method. Levels of p38β expression in WT neurons were normalized to 1.0.

### Primary Neuronal Culture

Primary neuronal cultures were derived from embryonic day 18 WT or p38β KO mice, as previously described (Xing et al. 2011). Cells were dissociated from dissected cerebral cortices by trypsinization for 20–25 min at 37 °C, followed by passing through a 70-μm nylon mesh cell strainer. The cells were seeded at a density of 5 × 10^4^ cells/well onto poly-d-lysine-coated 12-mm glass coverslips for L-glutamate and OGD experiments, or at 2 × 10^4^ cells/well in 24-well plates for SNP experiments. Neurons were grown in neurobasal medium containing 2 % B27 supplement (Invitrogen), 0.5 mM l-glutamine, 100 IU/ml penicillin, and 100 μg/ml streptomycin; no serum or mitosis inhibitors were used. Every 3 days, 50 % of the media was replenished with fresh medium.

### Cell Culture Treatments

Neurons from WT and p38β KO mice were subjected to L-glutamate, SNP, or OGD insults at 7 days in vitro (DIV7), and neurotoxicity measured at 24 h after insult. For L-glutamate studies, neurobasal/B27 medium was carefully removed from primary neuron cultures and saved. Neurons were then treated with 25 μM L-glutamate for 10 min in CSS buffer (120 mM NaCl, 5.4 mM KCl, 0.8 mM MgCl_2_, 1.8 mM CaCl_2_, 20 mM HEPES, and 15 mM glucose) (Schubert & Piasecki 2001). The cells were then washed three times with Hank’s balanced salt solution (HBSS), and returned back into the original neurobasal/B27 media for 24 h. WT neurons were treated with the p38α inhibitors MW-181 or MW-108 (60 μM) for 60 min before L-glutamate addition. For SNP studies, neurons were treated with 1 mM SNP dissolved in culture medium for 24 h before neurotoxicity assays. MW-181 or MW-108 (60 μM) was added at the same time as the SNP solution. For OGD studies, primary neurons were treated with the p38α inhibitor MW-181 or MW-108 (60 μM) for 60 min prior to OGD. OGD was done for 1 h in an anaerobic chamber saturated with 5 % CO_2_ and 95 % N_2_ in glucose-free DMEM medium. The OGD condition was terminated by switching cells back to normal culture conditions and incubating for 24 h until neurotoxicity assays were done. Control cells were incubated in DMEM with glucose in a normoxic incubator for the same period.

### Neuronal Viability Assay

Neuron viability was assayed by trypan blue exclusion (Xie et al. 2004). Neuron-containing coverslips were incubated with 0.2 % trypan blue in HBSS for 2 min in a 37 °C incubator and then gently rinsed three times with HBSS. Neurons were viewed under bright field microscopy at × 200 final magnification. Five to eight fields were chosen randomly per coverslip, and a total of 485 to 761 cells were counted per coverslip. Trypan blue-positive and negative neurons were counted per field and the ratio of positive cells to the total cells was taken as the percent neuronal death.

### Immunocytochemistry

Cells were fixed with 3.7 % formaldehyde containing 0.1 % Triton X-100 in PBS for 10 min at room temperature. After washing three times with PBS, the coverslips were incubated with blocking buffer (PBS containing 6 % goat serum, 3 % bovine serum albumin (fraction V), 0.1 % Triton X-100) for 30 min at room temperature. Primary chicken anti-MAP2 antibody (1:1,000, Neuromics, Cat. no. CH22103) was diluted in blocking buffer and incubated with the cells at room temperature for 2 h. For detection of MAP2 staining, the cells were incubated with secondary biotin SP-conjugated goat antichicken antibody (1:1,000, Jackson ImmunoResearch) for 1 h, followed by streptavidin Alexa Fluor® fluorescent 488 (1:1,000, Invitrogen) incubation in blocking buffer at room temperature for 1 h. Wide field fluorescent photomicrographs were obtained using a Nikon Eclipse Ti microscope with an Axiocam MRc5 digital camera (Carl Zeiss).

### Semi-automated Sholl Analysis

The semi-automated Sholl assay was used to measure the neurite degeneration of MAP2-labeled neurons, essentially as we previously described with a manual Sholl analysis (Xing et al. 2011). The original images were binarized and thresholded using NIH ImageJ. Sholl semi-automated analysis program was loaded from ImageJ plugins (http://imagej.nih.gov/ij/plugins/). The central point on the soma of each neuron was selected, and a series of concentric circles were drawn automatically, with the radius of the smallest sampling circle at 8 μm from the central point and the radius of the largest sampling circle at 50 μm with a radius step size of 0.167 μm. The Sholl analysis then determined how many times the neurites intersected the sampling circles, and measured the average intersections over the whole area occupied by the neurite per neuron. The mean of average intersections of 107–188 neurons per group was calculated, and the mean from control group was normalized to 0 % damage.

### Statistics

Statistical analysis was conducted using GraphPad prism software V.6 (GraphPad Software). Unless otherwise indicated, values are expressed as mean ± SEM. Groups of two were compared by unpaired *t* test. One-way ANOVA followed by Bonferroni’s multiple comparison test was used for comparisons among three or more groups. Statistical significance was defined as *p* < 0.05.

## Results

### Validation of p38β KO in Primary Cortical Neurons

**Fig. 1:**
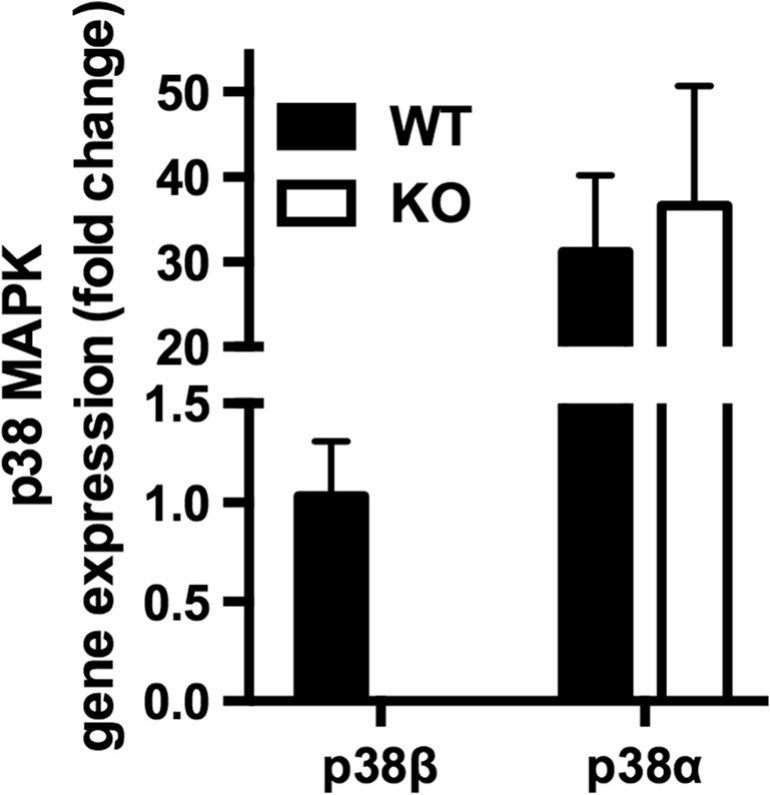
Verification of p38β KO in neurons. Primary cortical neurons from WT and p38β KO mice were prepared as described in the “Methods” section and plated at 5 × 10^4^ cells/well in 24-well plates. Total RNA was isolated from neuronal cultures derived from WT (*black bars*) or p38β KO (*white bars*) mice, and the mRNA levels of different p38 MAPK isoforms were determined by qPCR. The result shows that p38β mRNA was readily measureable in WT mice but was not detected in the p38β KO mice. The p38α MAPK isoform in both WT and p38β KO neurons was expressed at much higher levels compared to p38β, but there was no significant difference between the levels of p38α in WT and p38β KO mice. The levels of p38δ and p38γ mRNA were very low to undetectable in both WT and p38β KO mice (data not shown). Results are expressed as fold change compared to p38β expression levels in WT neurons, and represent the mean ± SEM of four to eight determinations

As a first step, it was important to confirm the deletion of p38β in primary cortical neurons from the p38β KO mouse and verify that significant compensatory changes in the p38α, p38δ, and p38γ isoforms were not present. RNA was prepared from primary cortical neuron cultures derived from WT or p38β KO mouse fetuses, and the expression levels of the p38 isoforms were determined by qPCR. As expected, p38β mRNA was readily measurable in WT mice but was not detected in the p38β KO mice (Fig. 1). The mRNA level of p38α in both WT and p38β KO neurons was ~40-fold higher than that of p38β in WT neurons, but there was no significant difference between the p38α levels in the WT compared to the p38β KO mice. The levels of p38δ and p38γ mRNA were similar and very low in both WT and p38β KO mice (data not shown). Altogether, the data verify that, as expected, p38β is deficient in neurons from the p38β KO mice and there are no significant compensatory changes in any of the other p38 isoforms.

### Neurotoxicity Induced by L-Glutamate

**Fig. 2:**
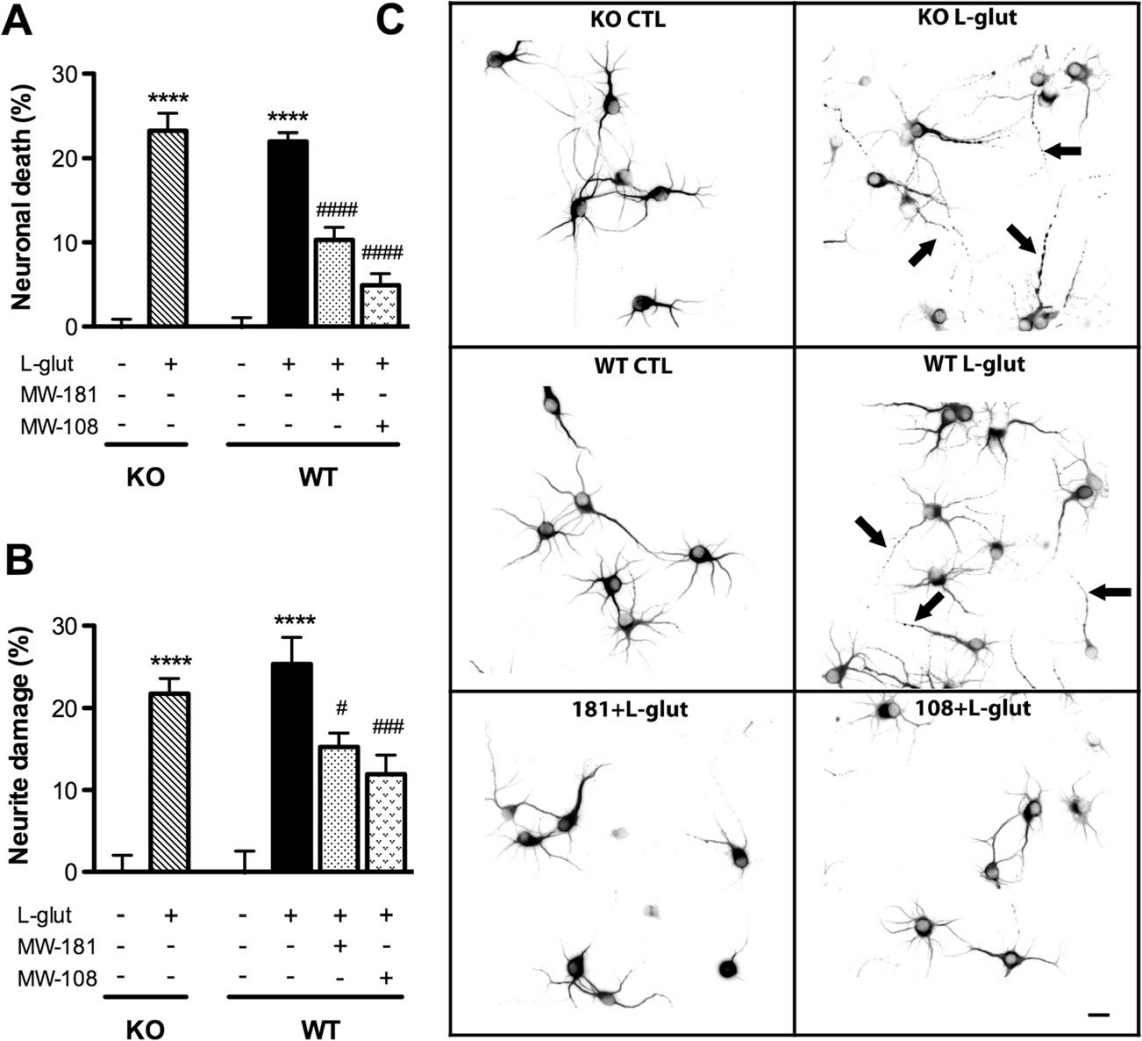
p38α inhibition but not p38β KO protects neurons against L-glutamate insult. WT or p38β KO mouse primary cortical neurons were plated on cover slips at 5 × 10^4^ cells/well and grown for 7 days in vitro (DIV7). After 1 h pretreatment of WT neurons with 60 μM MW-181 or MW-108, the media was removed and saved, then WT and p38β KO neurons were treated for 10 min with culture medium alone, L-glutamate (25 μM) alone, or L-glutamate plus 60 μM MW-181 or MW-108. After 10 min of incubation, cells were washed three times with HBSS, and the original culture media was added back into the appropriate wells. Trypan blue exclusion assay for neurotoxicity and Sholl analysis for neurite damage were performed after 24 h. **a** L-glutamate induced ~22 % neuronal death in both p38β KO and WT neurons. In contrast, p38α inhibition by MW-181 or MW-108 significantly reduced the neuron death after L-glutamate insult. **b** Similarly, L-glutamate-induced neurite fragmentation and blebbing in both p38β KO and WT neurons, with no significant difference between the two groups. In contrast, inhibition of p38α MAPK by MW-181 or MW-108 significantly protected neurites against L-glutamate-induced damage. **c** Representative photomicrographs of MAP2 immunocytochemistry show the morphology of neurons after 24 h. *Arrows* point to the appearance of damaged neurites after L-glutamate insult in both p38β KO and WT neurons (****p < 0.0001 vs. control; #*p* < 0.05 vs. L-glutamate treatment; ###*p* < 0.001 vs. L-glutamate treatment; ####*p* < 0.0001 vs. L-glutamate treatment, Bonferroni’s multiple comparison test). Data are from three independent experiments. *Scale bar* 10 μm

L-glutamate is a standard neurotoxic stimulus that is a model of excitotoxic cell death (Choi et al. 1987), and p38 has been reported to be involved in excitotoxic pathways leading to neuron damage/death (Kawasaki et al. 1997; Chen et al. 2003; Pi et al. 2004; Chaparro-Huerta et al. 2008; Molz et al. 2008; Bakuridze et al. 2009; Izumi et al. 2009). Therefore, we compared the degree of neuron death and neurite degeneration induced by L-glutamate in primary cortical neurons derived from WT and p38β KO mice. Under the culture conditions used, L-glutamate induced ~22 % neuron death as measured by trypan blue assay (Fig. 2a). L-glutamate also induced significant (22–25 %) neurite damage in the surviving neurons as measured by Sholl analysis (Fig. 2b), where the percentage of average intersections over the whole area occupied by the neurite is determined. L-glutamate treatment resulted in extensive neurite fragmentation, swelling, and blebbing (Fig. 2c). The degree of neuron death/neurite damage was not significantly different between WT and p38β KO neurons. In contrast, inhibition of neuronal p38α by MW-181 or MW-108 1 h prior to L-glutamate treatment significantly reduced both the neuron death and the neurite degeneration (Fig. 2a, b). As shown in Fig. 2c, the neurons treated with MW-181 or MW-108 showed less fragmentation and blebbing of the neurites.

### Neurotoxicity Induced by SNP

**Fig. 3:**
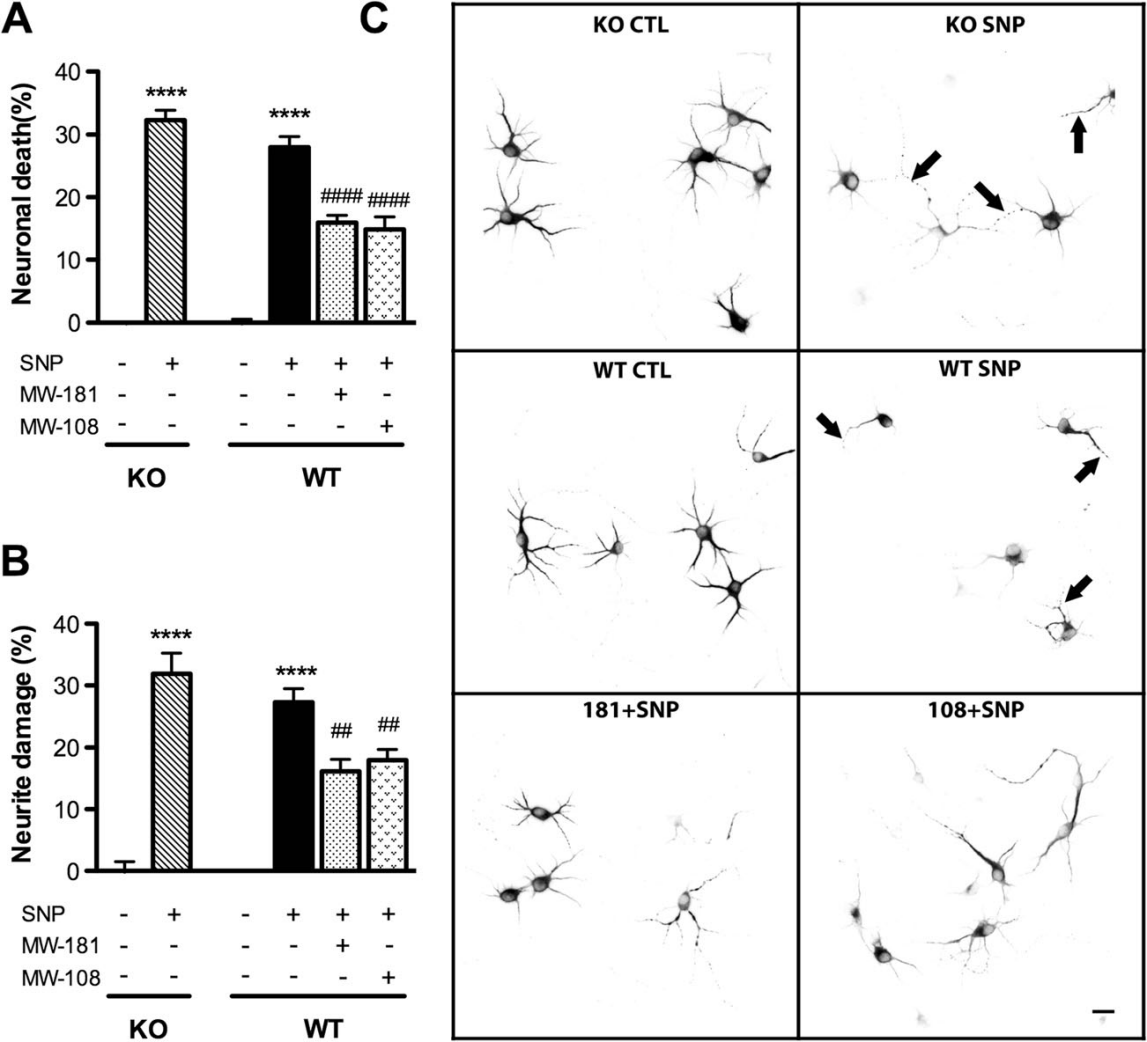
p38α inhibition but not p38β KO protects neurons against SNP insult. DIV7 neurons on coverslips were treated with culture medium alone, SNP (1 mM) alone, or SNP plus 60 μM MW-181 or MW-108 for 24 h, followed by trypan blue exclusion assay and Sholl analysis. **a** SNP induced ~28–32 % neuronal death in both p38β KO and WT neurons, with no significant differences between the genotypes. In contrast, p38α inhibition by MW-181 or MW-108 significantly reduced the neuron death induced by SNP. **b** SNP induced a similar degree of neurite damage in both p38β KO and WT neurons. In contrast, WT neurons treated with SNP in the presence of the p38α inhibitors showed reduced levels of neurite degeneration. **c** Representative photomicrographs of MAP2 immunocytochemistry show the morphology of neurons after 24 h. *Arrows* point to the appearance of damaged neurites induced by SNP treatment in both p38β KO and WT neurons (****p < 0.0001 vs. control; ##*p* < 0.01 vs. SNP; ####*p* < 0.0001 vs. SNP, Bonferroni’s multiple comparison test). Data are from three independent experiments. *Scale bar* 10 μm

To determine whether the findings with L-glutamate implicating p38α but not p38β in neurotoxicity were generalizable to a different neurotoxic insult, we tested the effect of SNP on neuron death and neurite damage. SNP is a nitric oxide donor commonly used to induce neuronal apoptosis, and p38 activation has previously been implicated in promoting nitric oxide induced neuronal damage (Ghatan et al. 2000; Lin et al. 2001). SNP (1 mM) treatment for 24 h killed 32 % WT neurons and 28 % p38β KO neurons (Fig. 3a) and induced 27–32 % neurite damage in both groups (Fig. 3b, c). Although the KO neurons appeared to be slightly less susceptible to SNP toxicity compared to WT neurons, the levels of neuron death/neurite damage between WT and p38β KO neurons were not significantly different. Similar to the findings with L-glutamate, inhibition of p38α by MW-181 or MW-108 treatments of WT neurons significantly reduced SNP-induced neuronal death (Fig. 3a), and protected neurons against neurite degeneration (Fig. 3b, c).

### Neurotoxicity Induced by OGD

**Fig. 4:**
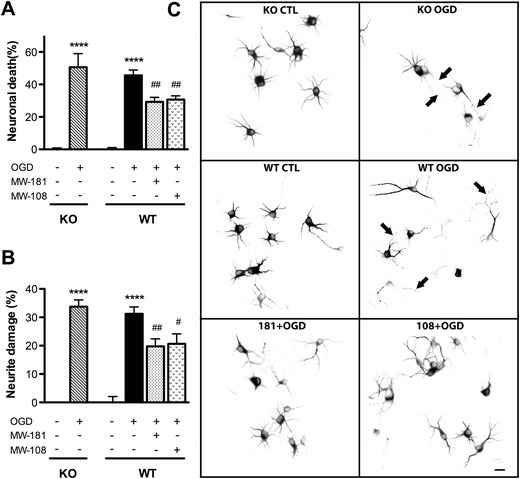
p38α inhibition but not p38β KO protects neurons against OGD insult. DIV7 neurons on coverslips were pretreated for 1 h with either 60 μM MW-181 or MW-108, and the medium was removed and saved. After 1 h OGD treatment, the old culture media was then added back into appropriate wells for 24 h, followed by measurement of neuronal survival and neurite damage. **a** OGD induced ~50 % neuronal death in both p38β KO and WT neurons, with no significant differences between the two groups. In contrast, p38α inhibition by MW-181 or MW-108 significantly reduced the neuronal death after OGD insult. **b** OGD induced a similar degree of neurite damage (~33 %) in both p38β KO and WT neurons. In contrast, p38α inhibition by MW-181 or MW-108 significantly protected neurites against OGD-induced damage. **c** Representative photomicrographs of MAP2 immunocytochemistry show the morphology of neurons after 24 h. *Arrows* point to the appearance of damaged neurites induced by OGD in both p38β KO and WT neurons (****p < 0.0001 vs. control; #*p* < 0.05 vs. OGD; ##*p* < 0.01 vs. OGD, Bonferroni’s multiple comparison test). Data are from three independent experiments. *Scale bar* 10 μm

We also tested the relative contribution of p38α and p38β to neurotoxic responses induced by OGD, a model of ischemic injury (Kaku et al. 1991; Dawson et al. 1996; Legos et al. 2002). Treatment with OGD for 1 h induced 45–50 % of neuron death measured at 24 h after insult, in both WT and p38β KO groups (Fig. 4a), and again no significant difference in the degree of cell death was found between these two groups. OGD treatment induced 30–34 % neurite damage in both groups (Fig. 4b, c), and there was no significant difference in the degree of neurite degeneration between WT and p38β KO neurons. Similar to the results with L-glutamate and SNP, treatment of WT neurons with MW-181 or MW-108 led to a significant reduction in the neuronal death (Fig. 4a) and neurite degeneration (Fig. 4b) induced by OGD. Again, the neurites in the compound-treated cultures appeared smoother and had more neurite branches compared to OGD treatment in the absence of compounds (Fig. 4c).

## Discussion

In this study, we tested the respective contribution of the p38α and p38β MAPK isoforms in the neurodegeneration induced by three neurotoxic insults, and addressed the question if targeting a single kinase is sufficient to provide neuroprotective effects. Our results demonstrate that targeting p38α MAPK in neurons provides significant protection against three different neurotoxic insults, while loss of neuronal p38β MAPK does not affect the neurodegenerative responses to any of the three insults. These findings complement and extend our previous studies (Xing et al. 2011; Xing et al. 2013) that documented the importance of glial p38α MAPK in stressor-induced proinflammatory cytokine production and microglia-mediated neuron death. Altogether, our data demonstrate key roles of p38α MAPK signaling in both glial and neuronal responses that are linked to neuronal dysfunction, and continue to indicate the potential of this kinase as a CNS drug discovery target.

A number of previous studies have suggested that activation of p38 MAPK signaling in neurons in response to disease-relevant cellular stressors contributes to neuron dysfunction and neuron death, and that inhibition of p38 MAPK in the neuron is neuroprotective. For example, the p38 MAPK pathway has been implicated in neuron death induced by a number of agents, including excitotoxic stimuli (Cao et al. 2004; Semenova et al. 2007; Chaparro-Huerta et al. 2008), nerve injury (Wang et al. 2005; Wittmack et al. 2005), hypoxia/ischemia (Wang et al. 2002; Guo & Bhat 2007), and potassium deprivation (Yeste-Velasco et al. 2009). Neuronal p38 MAPK has also been reported to be involved in diabetic neuropathy (Sweitzer et al. 2004), hyperpolarization-activated and voltage-gated channel activation after injury (Wittmack et al. 2005; Wynne 2006), neurofilament pathology in amyotrophic lateral sclerosis (Ackerley et al. 2004), hyperalgesia and spinal pain (Svensson et al. 2005), activity-induced dendritic spine reduction (Sugiura et al. 2009), kainite-induced seizures and neuronal damage (Namiki et al. 2007), presynaptic serotonin transporter activity (Zhu et al. 2006), and various cytokine-mediated neuronal damage responses (Li et al. 2003; Wang et al. 2005; Chaparro-Huerta et al. 2008; Xing et al. 2011). Almost all the mechanistic data supporting the role of p38 in neuron dysfunction has been generated using small molecule p38 inhibitors such as SB203580. The commercial availability of SB203580 has led to its widespread use; however, SB203580 is not selective for the p38α versus p38β isoform or even for the p38 family alone. SB203580 and second-generation SB compounds such as SB202190 inhibit multiple other kinases, including casein kinase-1 delta, glycogen synthase kinase-3beta, protein kinase A, receptor interacting protein-2, and cyclin G-associated kinase (Clerk & Sugden 1998; Lali et al. 2000; Godl et al. 2003; Bain et al. 2007). Thus, despite the extensive evidence provided by work using SB compounds that inhibiting p38 is neuroprotective, the relative role of p38α and p38β in the neuroprotective responses and whether targeting a single kinase (p38α or p38β) is sufficient to exert the neuroprotective effects had not been tested. To address these important questions, we utilized our recently developed, highly selective p38α inhibitors, MW-181 and MW-108 (Watterson et al. 2013), as well as a global p38β knockout mouse. The use of these reagents in primary cortical neuron cultures allowed us to directly demonstrate for the first time the involvement of neuronal p38α, and not p38β, in the neurotoxic responses to glutamate, SNP, and OGD.

Glutamate is a major CNS excitatory neurotransmitter, but excessive glutamate release and overstimulation of glutamate receptors can induce excitotoxic neuron death. Activation of neuronal p38 MAPK signaling is a well-characterized response to glutamate insult, but few previous studies have explored the importance of p38α versus p38β in excitotoxic neuron death. One relevant study (Cao et al. 2004) implicated p38α in glutamate-induced damage of primary cerebellar granule neurons in culture through the use of a dominant-negative p38α construct, but did not explore p38β involvement because no p38β was detected in the cultured neurons. Our results demonstrating the involvement of p38α in primary cortical neurons are consistent with this study, and also show that p38β is not required for glutamate-induced neuron death.

Nitric oxide overproduction has been linked to neuron death in acute and chronic neurological disorders (Dawson & Dawson 1996; Dawson et al. 1996; Lee et al. 1999; Sattler et al. 1999; Arundine & Tymianski 2004). Several studies have utilized nitric oxide donors as a neurotoxic stimulus and p38 MAPK inhibitors such as SB203580 to explore the role of p38 MAPK in mediating neurodegenerative responses of cultured neurons to nitrosative stress. In general, these studies have demonstrated neuroprotection against nitric oxide insult, through several proposed mechanisms including reduced mitochondrial dysfunction and inhibition of peroxynitrite/reactive oxygen species formation (Ghatan et al. 2000; Lin et al. 2001; Bossy-Wetzel et al. 2004; Thomas et al. 2008; Nashida et al. 2011). However, as far as we are aware, no previous study tested specific isoforms of p38 MAPK in the neurotoxic responses.

We also investigated the role of p38 MAPK in neurotoxicity induced by OGD, a model of hypoxia-ischemia. Several previous reports have implicated p38 MAPK signaling in OGD-induced neurotoxicity through the use of the multi-kinase SB family of inhibitors. For example, SB239063 protects neuron-enriched forebrain cultures against OGD insult (Legos et al. 2002), SB203580 reduces OGD-induced death in PC12 cells (Li et al. 2009), and SB203580 or expression of an antisense p38 MAPK construct only in neuronal cells reduces oxidative stress and neuron death in hippocampal slice cultures (Lu et al. 2011). Importantly, a seminal paper (Guo & Bhat 2007) used p38 isoform-specific siRNAs to show that p38α and not p38β was a major contributor to OGD-induced death in the NSC34 motoneuron cell line. Our results reported here using highly specific p38α inhibitors in WT primary cortical neuron cultures and using neurons cultured from the p38β global knockout mouse are consistent with that study, and extend the results to primary neurons.

Although the dispensable role of p38β MAPK in cortical neurons in our study might be attributed to its relatively low expression in these cells compared to the expression of p38α MAPK, the data are consistent with our previous study showing that p38β KO microglia did not provide neuroprotection for co-cultured WT neurons upon lipopolysaccharide treatment (Xing et al. 2013). We did not explore other potential mechanisms or cell types where p38β MAPK may contribute. However, some studies have suggested that p38β MAPK may be more important in glial cells, rather than neurons. For example, studies using transient global ischemia, transient focal ischemia, and kainic acid-induced seizure models all showed a delayed activation of astrocytes with p38β MAPK immunoreactivity, but not p38α (Che et al. 2001; Piao et al. 2002; Piao et al. 2003). In addition, p38β was upregulated after injury in different cell types with different temporal profiles, with an early and transient induction of p38β in neurons, followed by a later and prolonged induction in astrocytes (Piao et al. 2003). Furthermore, the strong substrate preference of ATF2 by p38β compared to p38α and differential regulation by upstream kinases (Jiang et al. 1996) also suggest that the two kinases may act on different downstream targets and exert different functions in response to injury. The available data suggest a more restricted repertoire of functions of p38β MAPK that might be cell-specific and signaling-specific temporally and spatially in the CNS.

It should be noted that our understanding of the role of neuronal p38α and p38β MAPK signaling in neurotoxic responses is in its infancy. From the literature, it appears that the quantitative importance of the p38 MAPK pathway relative to other stress-induced signaling pathways can vary depending on the cell type, developmental status, toxic stimulus, timing of activation, and cell-cell interactions. For example, even in the same neuronal cell type at the same developmental stage, the involvement of p38 can be dependent on the neurotoxic stimulus. Specifically, p38 was reported to be involved in glutamate-induced death of cerebellar granule neurons, whereas death induced by withdrawal of trophic support involved JNK but not p38 (Cao et al. 2004). It is also clear that multiple signaling pathways can be induced in response to specific stimuli, and therefore the importance of one particular pathway may depend on the time points analyzed. Another important consideration is that glial p38 signaling in response to toxic stimuli can affect neuronal viability (Izumi et al. 2009; Xing et al. 2011), which can complicate the interpretation of results in slice cultures or in vivo models. Finally, discrepant results could also be due to technical issues, such as different culture conditions, animal strains, type or age of neurons, and/or stimulus paradigm. For example, the expression of the glutamate NMDA receptor subunit NR1 in neurons cultured for DIV7 is less than that in DIV11 neurons (Schubert & Piasecki 2001), the neuronal death induced by SNP is increased in DIV21 versus DIV14 neurons (Dawson et al. 1993), and hippocampal neurons are more vulnerable than cortical neurons to OGD treatment (Jiang et al. 2004). Nevertheless, even with the above caveats, our results using three different neurotoxic insults in the same type (primary cortical neurons) and age (DIV7) cultures clearly document that suppression of p38α with highly specific kinase inhibitors provides neuroprotection whereas lack of p38β in the knockout mouse has no effect. The availability of these reagents should allow future exploration of the importance of p38 MAPK signaling in other models of neuronal death.

## Conclusions

Activation of neuronal p38 MAPK occurs in response to a number of disease-relevant stressors, and pharmacological inhibition of p38 MAPK is neuroprotective in both cell and animal models. However, the relative contribution of neuronal p38α and p38β to neurodegenerative responses had not been addressed previously. In this study, we used p38α- and p38β-specific reagents to demonstrate that inhibition of neuronal p38α provides significant neuroprotection against three different toxic insults, but that loss of neuronal p38β has no effect. These results demonstrate isoform-specific functions of these p38 kinases in the neuron, and support an important role of the p38α isoform in neurodegenerative responses to injury.
